# Editorial: Neuroglia Molecular Mechanisms in Psychiatric Disorders

**DOI:** 10.3389/fnmol.2018.00407

**Published:** 2018-10-31

**Authors:** Caterina Scuderi, Mami Noda, Alexei Verkhratsky

**Affiliations:** ^1^Department of Physiology and Pharmacology “Vittorio Erspamer”, SAPIENZA University of Rome, Rome, Italy; ^2^Laboratory of Pathophysiology, Graduate School of Pharmaceutical Sciences, Kyushu University, Fukuoka, Japan; ^3^Faculty of Biology, Medicine and Health, The University of Manchester, Manchester, United Kingdom; ^4^Faculty of Health and Medical Sciences, Center for Basic and Translational Neuroscience, University of Copenhagen, Copenhagen, Denmark; ^5^Achucarro Centre for Neuroscience, IKERBASQUE, Basque Foundation for Science, Bilbao, Spain

**Keywords:** neuroglia, astrocyte, microglia, oligodendrocyte, psychiatric disorder

Neuroglia are an extended class of cells of ectodermal (astroglia, oligodendroglia, and peripheral glial cells) and mesodermal (microglia) origin, which provide extensive homeostatic support, protection, and defense of nervous tissue (Kettenmann and Ransom, 2013; Verkhratsky and Butt, 2013; Verkhratsky and Nedergaard, 2018). Pathological potential of glial cells has been recognized since their discovery (Virchow, 1858; Andriezen, 1893; Nissl, 1899; Alzheimer, 1910), while recent decade highlighted the fundamental role of neuroglia in the progression of all types of neurological diseases (Giaume et al., 2007; Verkhratsky et al., 2013, 2014, 2016, 2017; Burda and Sofroniew, 2014; Sofroniew, 2014; Zeidan-Chulia et al., 2014; Burda et al., 2016; Pekny et al., 2016; Verkhratsky and Parpura, 2016; Ferrer, 2018). The neurogliopathology is complex and is represented by glial reactivity (activation of microglia, astrogliosis) or glial degeneration, atrophy, and loss of functions. In particular glial cells undergo prominent changes in the context of neuropsychiatric diseases; these changes are generally represented by loss and asthenia of astroglia and oligodendroglia in conjunction with microglial activation (Rajkowska and Stockmeier, 2013; Verkhratsky et al., 2015; Cobb et al., 2016; Rajkowska et al., 2018). In this research topic we addressed the contribution of neuroglia to major neuropsychiatric conditions including major depression, schizophrenia, and alcohol use disorders.

The gliocentric theory of major depression has been overviewed by Czéh and Nagy who presented recent findings on glial structural and functional abnormalities at molecular and cellular levels. Subsequently they focused on clinical studies and summarized neuroimaging as well as post-mortem molecular and histopathological evidence supporting leading role of glia in the progression of depression and related mood disorders. Notably, the depression is associated with substantial loss of macroglial cells with loss of their function, in conjunction with microglial activation, which summarily affect neuronal networks and brain function. Structural and molecular alterations of astrocytes and oligodendrocytes also lie at the core of alcohol use disorders (Miguel-Hidalgo). In particular alcohol exposure, as well as alcohol withdrawal affects glial transporters, glutamate (GABA) glutamine shuttle and gap junctions, which connect glial syncytia. The association of endothelial cells, blood-brain barrier and neurovascular unit with neuropsychiatric diseases has been discussed by Malik and Di Benedetto, who presented evidence supporting pathophysiological role of disrupted erythropoietin-producing-hepatocellular carcinoma receptors (EphR)/Ephrin signaling cascades.

Pathophysiological changes in microglia in context of environmental stress associated with neurodevelopmental and neuropsychiatric disorders are overviewed by Tay et al. In particular, they discuss the role of microglia in Nasu-Hakola disease, hereditary diffuse leukoencephaly with spheroids, Rett syndrome, autism spectrum disorders, obsessive-compulsive disorder and major psychiatric pathologies such as addiction, major depressive disorder, bipolar disorder, schizophrenia, eating disorders, and sleep disorders. Studies on humans have demonstrated neuroinflammation and microglial reactivity in patients with schizophrenia as well as in animal models. In the latter, the gender differences have been also described (Hui et al.); and it turned out that in males prominent differences in microglial distribution, morphology, and microglial activation may account for higher incidence of schizophrenia. Up-regulation of microglia associated genes following administration of IgG-Saporin has been demonstrated to alter a memory-associated behavior (Dobryakova et al.). The role of microglial activation and phagocytosis in Parkinson's disease and therapeutic potential of targeting microglia were reviewed by Janda et al. Of note both asphyxia and cesarean section are associated with the expression of Schizophrenia risk genes (Paparelli et al.). The link between neuregulin and β-secretase with schizophrenia is shown by Zhang et al.

Glial cells can be targets for specific therapy. Effects of electro-acupuncture on hippocampal neuroinflamamtion in rat depressed model (induced by unpredictable stress-induced depressive- and anxiety-like behavior) are presented by Yue et al. They showed that treatment with acupuncture reduce behavioral abnormalities and abrogate neuroinflammation. Quercitin neuroprotection was discussed by Gao et al.

All in all, the papers assembled in this Research Topic provide a wide picture of the importance of neuroglia in the pathogenesis of neuropsychiatric diseases and discuss possible therapeutic strategies aimed at glial cells.

## Author contributions

CS, MN, and AV wrote the editorial. All authors read and approved the submitted version.

### Conflict of interest statement

The authors declare that the research was conducted in the absence of any commercial or financial relationships that could be construed as a potential conflict of interest.

## References

[B1] Alzheimer A. (1910). Beiträge zur Kenntnis der pathologischen Neuroglia und ihrer Beziehungen zu den Abbauvorgängen im Nervengewebe, in Histologische und histopathologische Arbeiten über die Grosshirnrinde mit besonderer Berücksichtigung der pathologischen Anatomie der Geisteskrankheiten, Vol. 3, eds Nissl F. Alzheimer A. (Jena: Gustav Fischer), 401–562.

[B2] Andriezen W. L. (1893). The neuroglia elements of the brain. Brit. Med. J. 2, 227–230. 20754383 10.1136/bmj.2.1700.227 PMC2422013

[B3] Burda J. E. Bernstein A. M. Sofroniew M. V. (2016). Astrocyte roles in traumatic brain injury. Exp. Neurol. 275(Pt 3), 305–315. 10.1016/j.expneurol.2015.03.020 25828533 PMC4586307

[B4] Burda J. E. Sofroniew M. V. (2014). Reactive gliosis and the multicellular response to CNS damage and disease. Neuron 81, 229–248. 10.1016/j.neuron.2013.12.034 24462092 PMC3984950

[B5] Cobb J. A. O'Neill K. Milner J. Mahajan G. J. Lawrence T. J. May W. L.. (2016). Density of GFAP-immunoreactive astrocytes is decreased in left hippocampi in major depressive disorder. Neuroscience 316, 209–220. 10.1016/j.neuroscience.2015.12.044 26742791 PMC4836620

[B6] Ferrer I. (2018). Astrogliopathy in Tauopathies. Neuroglia 1, 126–150. 10.3390/neuroglia1010010

[B7] Giaume C. Kirchhoff F. Matute C. Reichenbach A. Verkhratsky A. (2007). Glia: the fulcrum of brain diseases. Cell Death Differ. 14, 1324–1335. 10.1038/sj.cdd.4402144 17431421

[B8] Kettenmann H. Ransom B. R. (2013). Neuroglia. Oxford: Oxford University Press.

[B9] Nissl F. (1899). Ueber einige Beziehungen zwischen Nervenzellerkrankungen und Erscheinungen bei verschiedenen Psychosen. Arch. Psychiatr. 32, 1–21.

[B10] Pekny M. Pekna M. Messing A. Steinhäuser C. Lee J. M. Parpura V.. (2016). Astrocytes: a central element in neurological diseases. Acta Neuropathol. 131, 323–345. 10.1007/s00401-015-1513-1 26671410

[B11] Rajkowska G. Legutko B. Moulana M. Syed M. Romero D. G. Stockmeier C. A.. (2018). Astrocyte pathology in the ventral prefrontal white matter in depression. J. Psychiatr. Res. 102, 150–158. 10.1016/j.jpsychires.2018.04.005 29660602 PMC6005746

[B12] Rajkowska G. Stockmeier C. A. (2013). Astrocyte pathology in major depressive disorder: insights from human postmortem brain tissue. Curr. Drug Targets 14, 1225–1236. 10.2174/13894501113149990156 23469922 PMC3799810

[B13] Sofroniew M. V. (2014). Multiple roles for astrocytes as effectors of cytokines and inflammatory mediators. Neuroscientist. 20:160–172. 10.1177/1073858413504466 24106265

[B14] Verkhratsky A. Butt A. M. (2013). Glial Physiology and Pathophysiology. Chichester: Wiley-Blackwell, 560.

[B15] Verkhratsky A. Marutle A. Rodríguez-Arellano J. J. Nordberg A. (2015). Glial asthenia and functional paralysis: a new perspective on neurodegeneration and alzheimer's disease. Neuroscientist 21, 552–568. 10.1177/1073858414547132 25125026

[B16] Verkhratsky A. Nedergaard M. (2018). Physiology of astroglia. Physiol. Rev. 98, 239–389. 10.1152/physrev.00042.2016 29351512 PMC6050349

[B17] Verkhratsky A. Parpura V. (2016). Astrogliopathology in neurological, neurodevelopmental and psychiatric disorders. Neurobiol. Dis. 85, 254–261. 10.1016/j.nbd.2015.03.025 25843667 PMC4592688

[B18] Verkhratsky A. Parpura V. Pekna M. Pekny M. Sofroniew M. (2014). Glia in the pathogenesis of neurodegenerative diseases. Biochem. Soc. Trans. 42, 1291–1301. 10.1042/BST20140107 25233406

[B19] Verkhratsky A. Rodríguez J. J. Parpura V. (2013). Astroglia in neurological diseases. Future Neurol. 8, 149–158. 10.2217/fnl.12.90 23658503 PMC3645493

[B20] Verkhratsky A. Steardo L. Parpura V. Montana V. (2016). Translational potential of astrocytes in brain disorders. Prog. Neurobiol. 144, 188–205. 10.1016/j.pneurobio.2015.09.003 26386136 PMC4794425

[B21] Verkhratsky A. Zorec R. Parpura V. (2017). Stratification of astrocytes in healthy and diseased brain. Brain Pathol. 27, 629–644. 10.1111/bpa.12537 28805002 PMC5599174

[B22] Virchow R. (1858). Die Cellularpathologie in ihrer Begründung auf Physiologische and Pathologische Gewebelehre. Zwanzig Vorlesungen gehalten während der Monate Februar, März und April 1858 im Pathologischen Institut zu Berlin. Berlin: Hirschwald, 440.

[B23] Zeidan-Chulia F. Salmina A. B. Malinovskaya N. A. Noda M. Verkhratsky A. Moreira J. C. (2014). The glial perspective of autism spectrum disorders. Neurosci. Biobehav. Rev. 38, 160–172. 10.1016/j.neubiorev.2013.11.008 24300694

